# *Populus trichocarpa* EXPA6 Facilitates Radial and Longitudinal Transport of Na^+^ under Salt Stress

**DOI:** 10.3390/ijms25179354

**Published:** 2024-08-29

**Authors:** Zhe Liu, Kexin Yin, Ying Zhang, Caixia Yan, Ziyan Zhao, Jing Li, Yi Liu, Bing Feng, Rui Zhao, Jian Liu, Kaiyue Dong, Jun Yao, Nan Zhao, Xiaoyang Zhou, Shaoliang Chen

**Affiliations:** 1 State Key Laboratory of Efficient Production of Forest Resources, College of Biological Science and Technology, Beijing Forestry University, Beijing 100083, China; liuz6415@163.com (Z.L.); ykx0303@126.com (K.Y.); zying@bjfu.edu.cn (Y.Z.); caixiayan2019@163.com (C.Y.); zzyan913@163.com (Z.Z.); lijing70747@163.com (J.L.); ly4862ccc@163.com (Y.L.); 18720795166@163.com (B.F.); ruizhao926@126.com (R.Z.); liujian20170703@163.com (J.L.); 18353541623@163.com (K.D.); zhaonan19880921@126.com (N.Z.); zhouxiaoyang@bjfu.edu.cn (X.Z.); 2 Guangdong Provincial Key Laboratory of Silviculture, Protection and Utilization, Guangdong Academy of Forestry, Guangzhou 510520, China; yaojun990@126.com

**Keywords:** *Populus*, expansin, cell wall loosening, root contractility, root extensibility, Na^+^ flux, photosynthesis, SOD, POD, CAT

## Abstract

Expansins are cell wall (CW) proteins that mediate the CW loosening and regulate salt tolerance in a positive or negative way. However, the role of *Populus trichocarpa* expansin A6 (PtEXPA6) in salt tolerance and the relevance to cell wall loosening is still unclear in poplars. *PtEXPA6* gene was transferred into the hybrid species, *Populus alba* × *P. tremula* var. *glandulosa* (84K) and *Populus tremula* × *P. alba* INRA ‘717-1B4’ (717-1B4). Under salt stress, the stem growth, gas exchange, chlorophyll fluorescence, activity and transcription of antioxidant enzymes, Na^+^ content, and Na^+^ flux of root xylem and petiole vascular bundle were investigated in wild-type and transgenic poplars. The correlation analysis and principal component analysis (PCA) were used to analyze the correlations among the characteristics and principal components. Our results show that the transcription of *PtEXPA6* was downregulated upon a prolonged duration of salt stress (48 h) after a transient increase induced by NaCl (100 mM). The *PtEXPA6*-transgenic poplars of 84K and 717-1B4 showed a greater reduction (42–65%) in stem height and diameter growth after 15 days of NaCl treatment compared with wild-type (WT) poplars (11–41%). The Na^+^ accumulation in roots, stems, and leaves was 14–83% higher in the transgenic lines than in the WT. The Na^+^ buildup in the transgenic poplars affects photosynthesis; the activity of superoxide dismutase (SOD), peroxidase (POD), and catalase (CAT); and the transcription of *PODa2*, *SOD [Cu-Zn]*, and *CAT1*. Transient flux kinetics showed that the Na^+^ efflux of root xylem and leaf petiole vascular bundle were 1.9–3.5-fold greater in the *PtEXPA6*-transgenic poplars than in the WT poplars. *PtEXPA6* overexpression increased root contractility and extensibility by 33% and 32%, indicating that *PtEXPA6* increased the CW loosening in the transgenic poplars of 84K and 717-1B4. Noteworthily, the *PtEXPA6*-promoted CW loosening was shown to facilitate Na^+^ efflux of root xylem and petiole vascular bundle in the transgenic poplars. We conclude that the overexpression of *PtEXPA6* leads to CW loosening that facilitates the radial translocation of Na^+^ into the root xylem and the subsequent Na^+^ translocation from roots to leaves, resulting in an excessive Na^+^ accumulation and consequently, reducing salt tolerance in transgenic poplars. Therefore, the downregulation of *PtEXPA6* in NaCl-treated *Populus trichocarpa* favors the maintenance of ionic and reactive oxygen species (ROS) homeostasis under long-term salt stress.

## 1. Introduction

The cell wall proteins expansins (EXPs) mediate cell wall loosening by breaking the hydrogen bonds between cellulose microfibrils and matrix polymers [1,2]. In addition to regulating wall extension during plant growth, expansins are also involved in plant responses to various environmental stresses such as cold, heat, drought, salt, heavy metal, and osmotic stress [3,4,5,6,7,8]. Much attention has been paid to the role of expansins in stress physiology, and there is now considerable experimental evidence that expansins may mediate the plant response to salinity. Salt induced the upregulation of wheat expansin genes in leaves (*TaEXPA3-A*, *TaEXPB2-A*, *TaEXPB4-A*, *TaEXPB10-A*, and *TaEXPA9-A*) and roots (*TaEXPB4-A*, *TaEXPB10-A,* and *TaEXPA6-A*) [9]. The overexpression of tobacco *NtEXPA4* was shown to confer salt and drought tolerance, while RNAi mutants exhibited increased hypersensitivity to salinity [10]. The grass *AstEXPA1* gene improved the performance of transgenic tobacco plants under salt stress [11]. Similarly, *AtEXP2* overexpression may confer greater tolerance to salt and osmotic stress in *Arabidopsis* seed germination [12]. Recently, the expansin gene *SmEXPA23* from *Salix matsudana* and the expansin gene *PttEXPA8* from *Populus tomentosa* also increased the salt tolerance of plants [13,14].

There is increasing evidence that expansins modulate morphological and anatomical features to confer salt tolerance in various species like *Nicotiana tabacum* (*Nt*), *Rosa hybrida* (*Rh*), and *Oryza sativa* (*Os*). *NtEXPA11*-overexpressed plants have significantly larger pith and parenchyma cells compared to the wild type (WT) [15]. The overexpression of *RhEXPA4* leads to several changes in the epidermal structure of leaves that respond to abiotic stress, e.g., smaller, compact cells, fewer stomata, and midvein vascular patterning in leaves [16]. In addition, *OsEXPA7* overexpression increases cell size and number in the leaf and the elongation of metaxylem cells in the root, which may be involved in improving CW loosening and salt tolerance [17]. Similarly, *RhEXPA4*-transgenic plants had more lateral roots and longer primary roots under salt stress [16]. It is assumed that the expansin-mediated CW loosening and cell elongation reduces Na^+^ concentration in the cytoplasm and vacuoles due to the increased water uptake [17]. Expansins have been shown to increase salt tolerance by improving the plant water status, ionic relationships, and ROS homeostasis under saline conditions. For example, the overexpression of *TaEXPB23* in tobacco improved salt tolerance by decreasing osmotic potential and increasing water retention ability [18]. The ectopic expression of wheat expansin gene *TaEXPA2* in tobacco improved salt tolerance by regulating water status, antioxidant defense, and Na^+^/K^+^ balance [19]. *Chenopodium quinoa* expansin 50 (CqEXPA50) could promote the accumulation of photosynthetic pigment and activate enzymatic and non-enzymatic antioxidant systems [20]. Similarly, *OsEXPA7* overexpression resulted in a reduction in Na^+^ and ROS, and an accumulation of K^+^ in the leaves and roots under salt stress [17].

In contrast to the increase in salinity tolerance, there is also evidence that expansins negatively influence the response of plants to salinity. The overexpression of two typical Arabidopsis α- and β-expansin genes, *AtEXP3* and *AtEXPβ1*, resulted in increased sensitivity to salt stress. Although an increased peroxidase activity was observed in both *AtEXP3*- and *AtEXPβ1*-overexpressed seedlings, their overexpression can lead to deleterious defects in growth and development [21]. It has also been shown that excessive *AtEXPA1* expression could disrupt cell wall organization and lead to growth reduction, which facilitates plant adaptation to environmental stress [22]. In addition, the expression of *Festuca arundinacea EXPA7* decreased by 74–82% in the fast-growing genotype ‘K-310’ and the slow-growing genotype ‘Bonsai’ under salt stress [23]. It is, therefore, possible that salinized plants downregulate the transcription of expansins to reduce CW loosening and plant growth under unfavorable conditions. However, this needs to be clarified by further investigations.

In this study, we report a downregulation of expansin A6 (EXPA6) in long-term salt-stressed *Populus trichocarpa*. The purpose of this study was to investigate whether *Populus trichocarpa* PtEXPA*6* mediates the salt tolerance of poplars and its relevance to cell wall loosening. *PtEXPA6* gene was transferred into the hybrid species, *Populus alba* × *P. tremula* var. *glandulosa* (84K) and *Populus tremula* × *P. alba* INRA ‘717-1B4’. Under salt stress, the stem growth, gas exchange, chlorophyll fluorescence, activity and transcription of antioxidant enzymes, Na^+^ content, and Na^+^ flux of root xylem and petiole vascular bundle were investigated in wild-type and transgenic poplars. The correlation analysis and principal component analysis (PCA) were used to analyze the correlations among the characteristics and principal components. Our results showed that the overexpression of *PtEXPA6* reduced plant growth, photosynthetic capacity, and the ability for ROS scavenging under salt stress, which was related to the excessive Na^+^ transported from root to shoots. The *PtEXPA6*-promoted cell wall loosening and the relevance to apoplastic Na^+^ transport were explored in the root tips of the WT and transgenic poplars. We hypothesize that the overexpression of *PtEXPA6* leads to loosening of the cell walls, facilitating the radial translocation of Na^+^ into the root xylem and the subsequent Na^+^ translocation from roots to leaves, resulting in an excessive Na^+^ accumulation and reduced salt tolerance in the transgenic poplars of 84K and 717-1B4.

## 2. Results

### 2.1. Expression of PtEXPA6 in Root and Shoot of Populus trichocarpa

**Figure 1:**
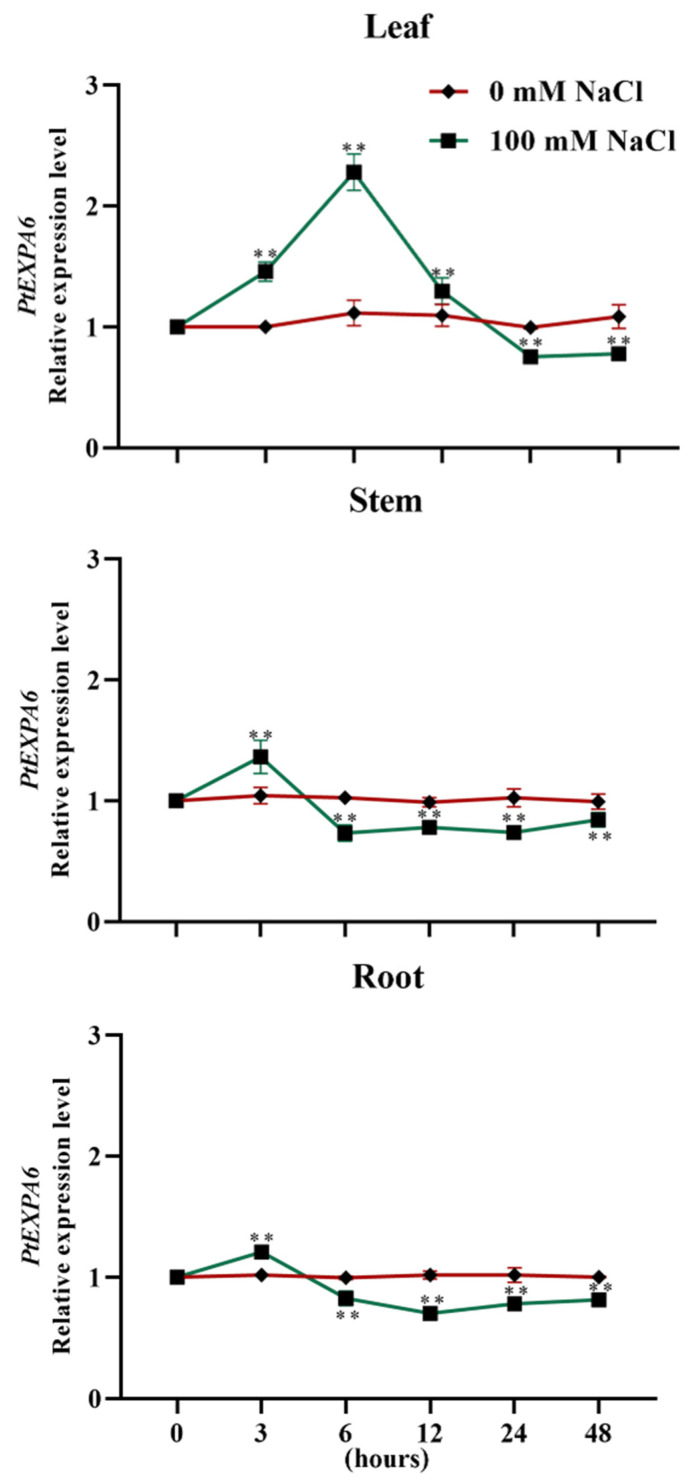
Transcription profile of *PtEXPA6* in the leaves, stems, and roots of *Populus trichocarpa* during the period of salt stress. Uniform plants of *P. trichocarpa* were treated with NaCl saline (0 or 100 mM) for 48 h. The fine roots, stems, and upper leaves (3rd to 8th from shoot tip) were sampled at 0, 3, 6, 12, 24, and 48 h, respectively. For the RT-qPCR analysis, the primer sequences for *PtEXPA6* and the reference gene, *PtUBQ*, are shown in Supplementary Table S1. The data are means ± SD (*n* = 3), and the bars with asterisks indicate significant differences, **: *p* < 0.01.

The transcription level of *PtEXPA6* in *P. trichocarpa* fluctuated during the observation period of salt stress (100 mM NaCl, 48 h). The expression of *PtEXPA6* was transiently upregulated in the leaves and peaked after 6 h of salt treatment, followed by a rapid decrease to a level below that of the controls after 24 h (Figure 1). The *PtEXPA6* expression then stabilized at the end of the salt treatment (48 h) and remained below the pre-treatment level (Figure 1). In the roots and stems, the NaCl-altered transcription of *PtEXPA6* was similar to that in the leaves, with an initial upregulation at 3 h, followed by a significant decrease at 6 h, and stabilized at a level lower than that of the controls (Figure 1). 

### 2.2. Homologous Sequence Analysis of PtEXPA6

**Figure 2:**
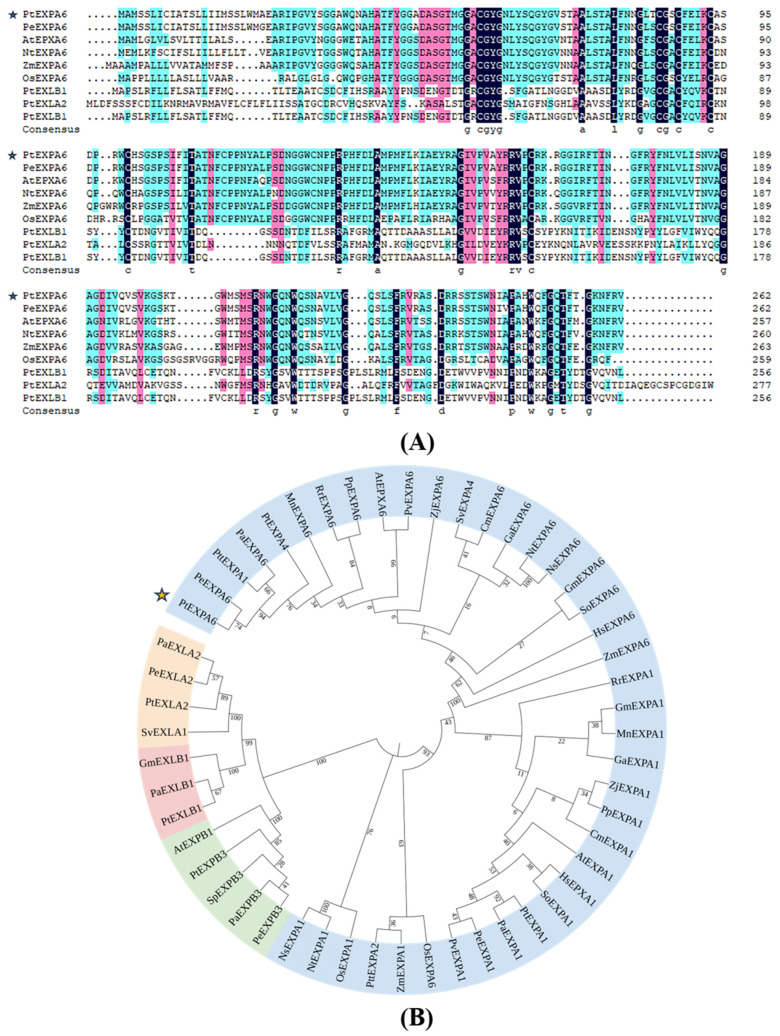
Sequence and phylogenetic analysis of *Populus trichocarpa PtEXPA6*. (**A**) The multiple sequence alignment of EXPA and expansin family from *Populus* and other species. The black shading indicates identical amino acid residues, and the blue and pink shadings indicate conserved amino acids, respectively. The lower-case letters represent the same amino acids in different species. (**B**) The phylogenetic analysis of expansin from various species. *Populus euphratica* (Pe), *Populus trichocarpa* (Pt), *Populus tremula × Populus tremuloides* (Ptt), *Populus alba* (Pa), *Arabidopsis thaliana* (At), *Zea mays* (Zm), *Nicotiana tabacum* (Nt), and *Oryza sativa* (Os), *Glycine max* (Gm), *Salix viminalis* (Sv), *Morus notabilis* (Mn), *Rosa rugosa* (Rr), *Prunus persica* (Pp), *Pistacia vera* (Pv), *Ziziphus jujuba* (Zj), *Cucumis melo* (Cm), *Gossypium arboreum* (Ga), *Nicotiana sylvestris* (Ns), *Syzygium oleosum* (So), *Hibiscus syriacus* (Hs), *Pistacia vera* (Pv), and *Salix purpurea* (Sp). Supplementary Table S2 lists the accession numbers of the EXPA orthologs. The blue, yellow, pink and green shadings indicate expansin orthologs of EXPA, EXLA, EXLB, and EXPB, respectively. The PtEXPA6 is labelled with star symbols in (**A**,**B**).

The coding sequence (CDS) of *PtEXPA6* is 789 bp in length, encoding 266 amino acids. The translated protein sequence has a molecular weight of 28.38 kDa and an isoelectric point of 9.79 (Figure 2A). The phylogenetic analysis revealed that PtEXPA6 is evolutionarily closely related to PeEXPA6 in *P. euphratica*, but more distantly related to AtEXPA6 in *Arabidopsis thaliana* (Figure 2B).

### 2.3. Overexpression of PtEXPA6 in Poplars

**Figure 3:**
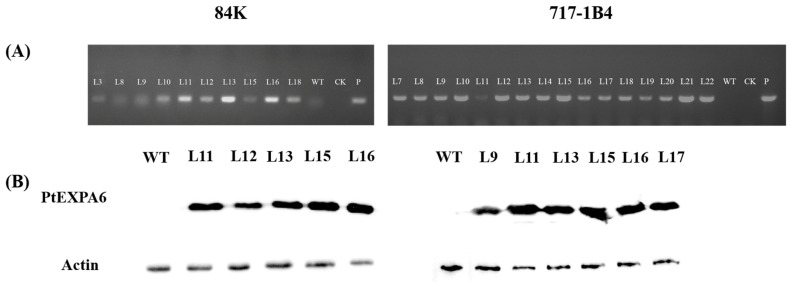
Molecular verification of the transgenic lines overexpressing *P. trichocarpa PtEXPA6* in 84K and 717-1B4. (**A**) The PCR assay of the transgenic poplars. The primer sequences for *PtEXPA6* are shown in Supplementary Table S1. WT: negative control (wild type); CK: blank control; P: positive control. (**B**) The Western blot of the transgenic lines. The Western blot analysis performed with an anti-MYC-specific antibody for PtEXPA6.

The downregulation of *PtEXPA6* following the NaCl treatment suggests that it is involved in the response of *P. trichocarpa* to salt stress. To investigate the regulatory effects of *PtEXPA6* under saline conditions, we overexpressed *PtEXPA6* in the hybrid poplars of 84K and 717-1B4. PCR assay revealed different expressions of *PtEXPA6* in the transgenic lines of 84K and 717-1B4 (Figure 3A). The Western blot analysis confirmed five transgenic lines of 84K and six transgenic lines of 717-1B4 with higher expression levels, three of which were selected for further investigation, i.e., L11, L12, and L13 for the 84K poplar and L9, L15, and L16 for the 717-1B4 poplar (Figure 3).

### 2.4. Effect of Salinity on Shoot Growth of PtEXPA6-Overexpressed Poplars

**Figure 4:**
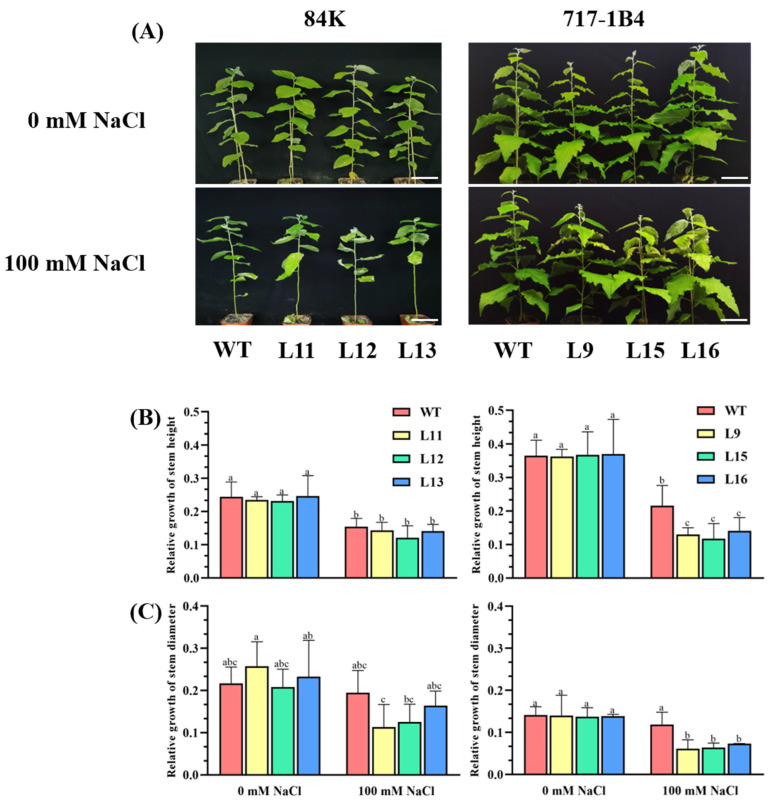
Phenotypic tests of the wild-type (WT) and *PtEXPA6*-overexpressing lines of 84K and 717-1B4 under long-term salt stress. The *PtEXPA6*-overexpressing lines of 84K (L11, L12, and L13) and 717-1B4 (L9, L15, and L16), and wild-type (WT) were exposed to NaCl with 0 or 100 mM for 15 days. The stem height and diameter of the no-salt control and salinized plants were measured after 15 days of the salt treatment. The relative growth of the stem height and diameter during the observation period are shown. (**A**) Representative images showing plant performance after the salt treatment. Scale bars = 5 cm. (**B**) Relative growth of the stem height. (**C**) Relative growth of the stem diameter. The data are means ± SD (*n* = 3), and the bars with different letters indicate significant differences (*p* < 0.05).

The NaCl effects on stem height and diameter growth were investigated by exposing the WT and *PtEXPA6*-overexpressed poplars to 100 mM NaCl for 15 days. Under normal growth conditions, there was no significant difference in the plant height and ground diameter between wild-type and transgenic lines (Figure 4A−C). However, the WT and transgenic lines of 84K and 717-1B4 showed significantly reduced growth after the salt treatment (*p* < 0.05), with the growth of the transgenic lines being more suppressed (Figure 4B,C). In comparison, the transgenic poplars of 717-1B4 showed a greater reduction in the plant height and ground diameter (52–65%) under salt stress than the transgenic poplars of 84K (42–43%, Figure 4).

### 2.5. Effect of Salinity on Photosynthesis of PtEXPA6-Overexpressing Poplars

**Figure 5:**
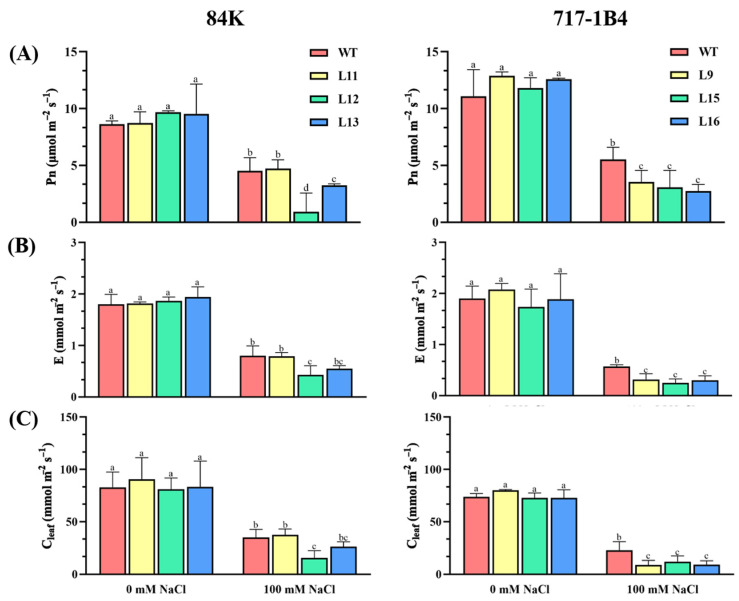
Effect of NaCl on leaf gas exchange in the wild-type and *PtEXPA6*-overexpressing lines of 84K and 717-1B4. The *PtEXPA6*-overexpressing lines of 84K (L11, L12, and L13) and 717-1B4 (L9, L15, and L16), and wild-type (WT) were exposed to NaCl with 0 or 100 mM for 15 days. Leaf gas exchange, i.e., net photosynthetic rate, transpiration rate, and stomatal conductance were measured in the leaves of the no-salt control and salinized plants after 15 days of the salt treatment. (**A**) Net photosynthetic rate (Pn). (**B**) Transpiration rate (E). (**C**) Stomatal conductance (Cleaf). The data are means ± SD (*n* = 3), and the bars with different letters indicate significant differences (*p* < 0.05).

**Figure 6:**
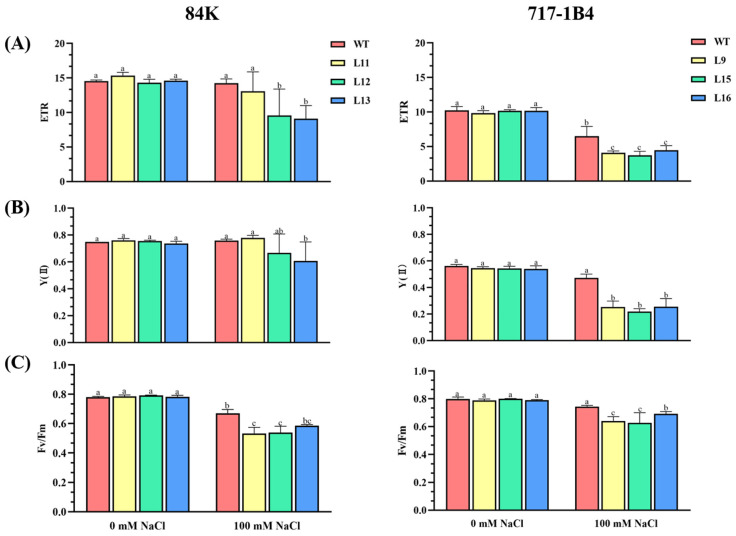
Effect of NaCl on chlorophyll fluorescence in the wild-type and *PtEXPA6*-overexpressing lines of 84K and 717-1B4. The *PtEXPA6*-overexpressing lines of 84K (L11, L12, and L13) and 717-1B4 (L9, L15, and L16), and wild-type (WT) were exposed to NaCl with 0 or 100 mM for 15 days. Chlorophyll fluorescence, i.e., the relative electron transport rate, the actual photosynthetic quantum yield, and the maximum photochemical efficiency of PSII were measured in the leaves of the no-salt control and salinized plants after 15 days of the salt treatment. (**A**) The relative electron transport rate (ETR). (**B**) The actual photosynthetic quantum yield (YII). (**C**) The maximum photochemical efficiency of PSII (Fv/Fm). The data are means ± SD (*n* = 3), and the bars with different letters indicate significant differences (*p* < 0.05).

The salt-restricted growth of the transgenic poplars was related to the reduced photosynthetic capacity. Leaf gas exchange and chlorophyll fluorescence were analyzed in the WT and transgenic lines of 84K and 717-1B4. After 15 days of salt stress, the net photosynthetic rate (Pn) decreased more in the transgenic lines of 84K (67%) and 717-1B4 (74%) compared to the WT poplars (46%, Figure 5A). The decrease in transpiration rate and stomatal conductance were also more pronounced in the transgenic lines than in the WT (Figure 5). Chlorophyll fluorescence measurements showed a decrease in the relative electron transport rate (ETR), actual photosynthetic quantum yield (YII), and maximum photochemical PSII efficiency (Fv/Fm) due to NaCl stress; however, the decrease was less pronounced in the WT (13%) compared to the transgenic poplars (16–44%), in particular, the 717-1B4 (Figure 6).

### 2.6. The Activity and Transcription of Antioxidant Enzymes 

**Figure 7:**
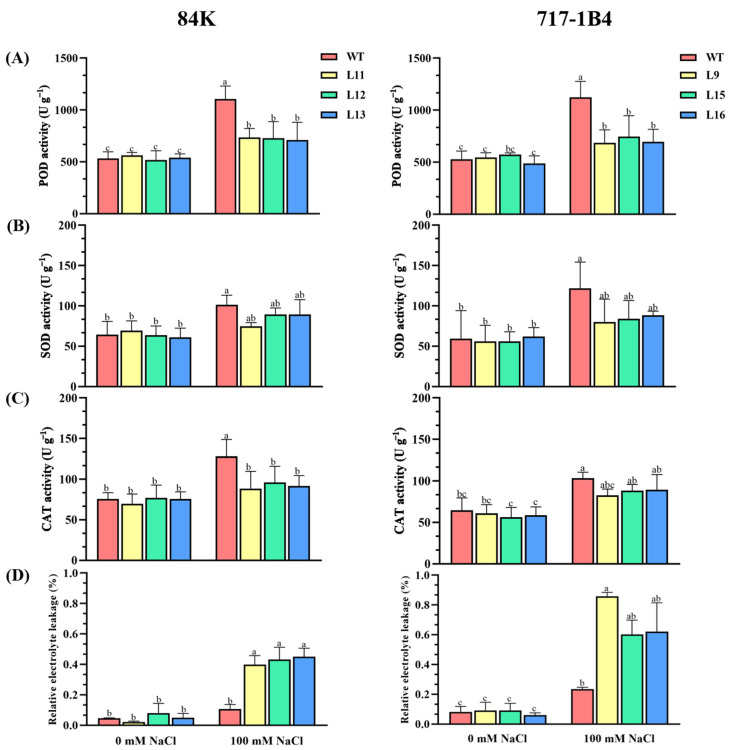
Effect of NaCl on antioxidant enzyme activity and relative electrolyte leakage in the wild-type and *PtEXPA6*-overexpressing lines of 84K and 717-1B4. The *PtEXPA6*-overexpressing lines of 84K (L11, L12, and L13) and 717-1B4 (L9, L15, and L16), and wild-type (WT) were exposed to NaCl with 0 or 100 mM for 15 days. The activity of superoxide dismutase (SOD), peroxidase (POD), and catalase (CAT), and relative electrolyte leakage were measured in the leaves of the no-salt control and salinized plants after 15 days of the salt treatment. (**A**) POD activity. (**B**) SOD activity. (**C**) CAT activity. (**D**) Relative electrolyte leakage. The data are means ± SD (*n* = 3), and the bars with different letters indicate significant differences (*p* < 0.05).

The ability to control ROS is critical for plant adaptation to saline environments. The activities of superoxide dismutase (SOD), peroxidase (POD), and catalase (CAT) were examined in the WT and transgenic lines of 84K and 717-1B4 under salt stress. The activities of the antioxidant enzymes increased significantly in all the genotypes tested under salt stress (*p* < 0.05), although the increase was 29–71% lower in the transgenic lines than in the WT (Figure 7A–C). In addition, the relative electrolyte leakage (REL) of each genotype was evaluated under control and salt treatments. The REL showed no significant difference between the genotypes tested under normal conditions, but a significant increase in the transgenic lines compared to WT after the salt treatment (*p* < 0.05, Figure 7D). 

**Figure 8:**
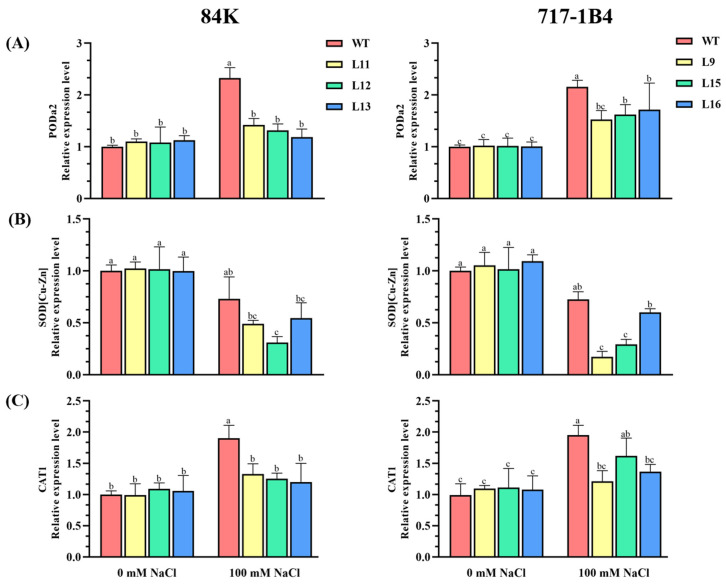
Effect of NaCl on the transcription levels of antioxidant enzymes in the wild-type and *PtEXPA6*-overexpressing lines of 84K and 717-1B4. The *PtEXPA6*-overexpressing lines of 84K (L11, L12, and L13), 717-1B4 (L9, L15, and L16), and wild-type (WT) were exposed to NaCl with 0 or 100 mM for 15 days. The relative expression of the antioxidant enzyme genes such as peroxidase a2 (*PODa2*), superoxide dismutase [Cu-Zn] (*SOD [Cu-Zn]*), and catalase 1 (*CAT1*) were examined in the WT and *PtEXPA6*-overexpressing poplars after 15 days of the salt treatment. (**A**) *PODa2*. (**B**) *SOD [Cu-Zn]*. (**C**) *CAT1*. The primer sequences of *PODa2*, *SOD [Cu-Zn]*, and *CAT1* and the reference actin gene, *PtUBQ*, are shown in Supplementary Table S1. The data are means ± SD (*n* = 3), and the bars with different letters indicate significant differences (*p* < 0.05).

In this study, we analyzed the transcription of *SOD [Cu-Zn]*, *CAT1*, and *PODa2* in the leaves of the WT and transgenic poplars. After the salt treatment, the transcription levels of *CAT1* and *PODa2* increased significantly in the WT poplars and the transgenic lines of 84K and 717-1B4 (*p* < 0.05), and the upregulation of *CAT1* and *PODa2* was 55–92% greater in the WT than the transgenic poplars (Figure 8). Conversely, the transcription level of *SOD [Cu-Zn]* was decreased in the salt-exposed poplars (Figure 8). And, the transgenic lines 84K and 717-1B4 showed a 29–38% greater decrease than the WT under the salt treatment (Figure 8).

### 2.7. Na^+^ Content in Roots, Stems, and Leaves of Transgenic Poplars

**Figure 9:**
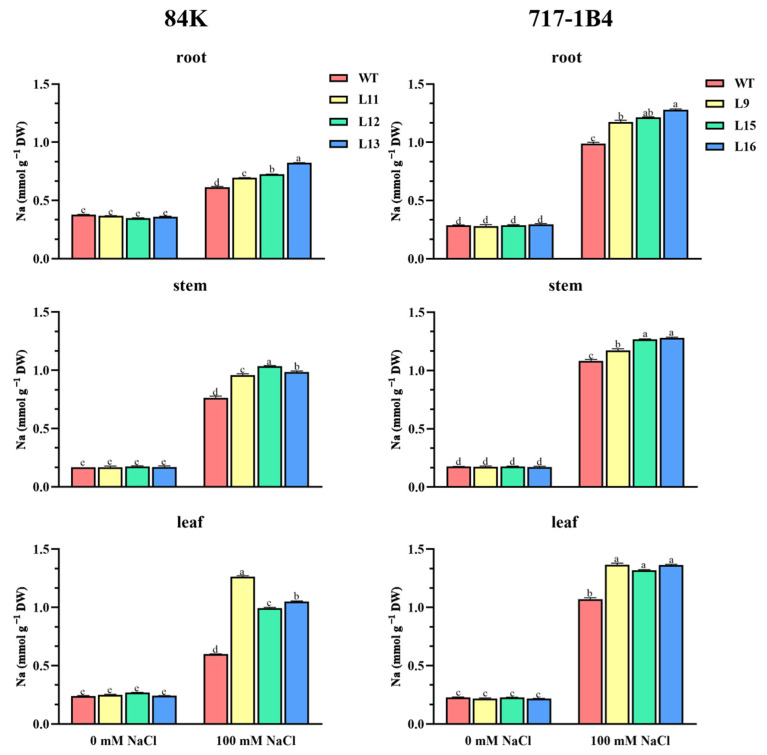
Na^+^ content in roots, stems, and leaves of wild-type and *PtEXPA6*-overexpressing lines of 84K and 717-1B4 under long-term salt stress. *PtEXPA6*-overexpressing lines of 84K (L11, L12, and L13) and 717-1B4 (L9, L15, and L16), and wild-type (WT) were exposed to NaCl with 0 or 100 mM for 15 days. Data are means ± SD (*n* = 3), and bars with different letters indicate significant differences (*p* < 0.05).

Maintaining the Na^+^ homeostasis is crucial for poplar adapting to salt stress [24]. The content of Na^+^ ions in roots, stems, and leaves was analyzed in the WT and transgenic poplars of 84K and 717-1B4. Under salt-free conditions, the Na^+^ content in the roots, stems, and leaves of the 84K poplar was low, with no significant difference between the WT and transgenic lines (Figure 9). However, the Na^+^ content increased significantly in the roots, stems, and leaves of 84K poplars after the salt treatment, and the *PtEXPA6*-trangenic lines, L11, L12, and L13, had 22–83% higher Na^+^ content than the WT (Figure 9). Similar trends were observed in the 717-1B4 poplar, where the Na^+^ content in the roots, stems, and leaves of the *PtEXPA6*-trangenic lines L9, L15, and L16 was 14–26% higher than that of the WT under salt stress (Figure 9). In comparison, the Na^+^ accumulation in the transgenic 717-1B4 poplar was 22–63% higher than in the transgenic poplar of 84K (Figure 9).

### 2.8. Comparative Contractability and Comparative Extensibility of Root Cell Walls 

**Figure 10:**
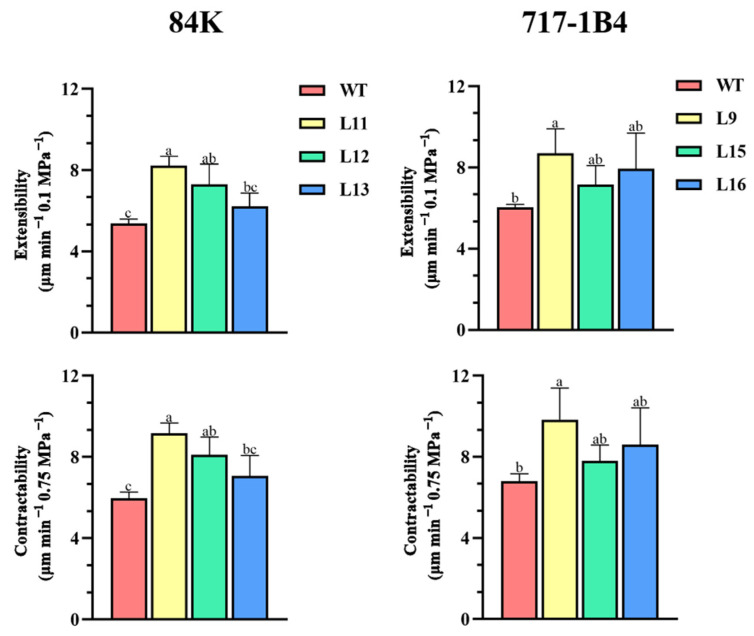
Comparative contractability and comparative extensibility of the intact root tip sites in the wild-type and *PtEXPA6*-overexpressing lines of 84K and 717-1B4. The comparative contractability of the *PtEXPA6*-overexpressing lines of 84K (L11, L12, and L13) and 717-1B4 (L9, L15, and L16), and wild-type (WT) was measured after the intact root tips were exposed to 300 mOsmol kg^−1^ mannitol (−0.75 MPa). Then, comparative extensibility of the intact root tip was measured after a 0.10 MPa osmotic jump. The data are means ± SD (*n* = 3), and the bars with different letters indicate significant differences (*p* < 0.05).

Expansins are cell wall (CW) proteins to mediate CW loosening by breaking the hydrogen bonds between cellulose microfibrils and matrix polymers [1,2]. The PtEXPA6-promoted CW loosening was determined by measuring comparative contractability and comparative extensibility using the intact root tips of the WT and transgenic poplars [25,26]. The comparative contractility of the intact root tips was measured after exposure to 300 mOsmol kg^−1^ mannitol treatment (−0.75 MPa^−1^) and expressed in µm min^−1^ 0.75 MPa^−1^. The comparative root extensibility values were obtained for the intact roots by determining the initial (1 min) increase in the root elongation rate induced by a 0.1 MPa osmotic jump [25,26]. Our results show that the overexpression of *PtEXPA6* in 84K and 717-1B4 led to an increased root contractability (8.1–8.7 μm min^−1^ 0.75 MPa^−1^) compared to the WT (6.0–6.8 μm min^−1^ 0.75 MPa^−1^). Moreover, *PtEXPA6* overexpression also increased the root comparative extensibility (7.3–7.9 μm min^−1^ 0.1 MPa^−1^) compared to the WT (5.4–6.0 μm min^−1^ 0.1 MPa^−1^) (Figure 10). Therefore, the elevated comparative contractability and comparative extensibility indicate PtEXPA6 increased cell wall loosening in the transgenic poplars.

### 2.9. Na^+^ Flux of Root Xylem and the Response to Osmotic Jump

**Figure 11:**
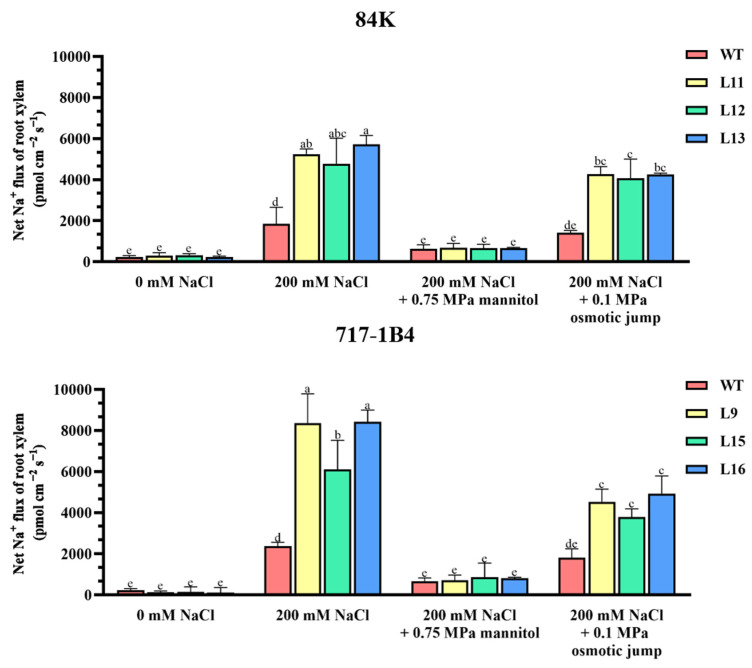
Na^+^ flux of the root xylem and the response to an osmotic jump in the wild-type and *PtEXPA6*-overexpressing lines of 84K and 717-1B4. After exposure to 200 mM NaCl for 4 h, the intact root tips of the *PtEXPA6*-trangenic lines of 84K (L11, L12, and L13) and 717-1B4 (L9, L15, and L16), and wild-type (WT) were exposed to 300 mOsmol kg^−1^ mannitol (−0.75 MPa), followed by a 0.1 MPa osmotic jump. The net Na^+^ flux of the root xylem was measured before and after the addition of mannitol and the subsequent 0.1 MPa osmotic jump. The data are means ± SD (*n* = 3), and the bars with different letters indicate significant differences (*p* < 0.05).

The greater Na^+^ accumulation in the transgenic poplars mainly results from the salt uptake and transport in roots and the root-to-shoot salt transport [24]. The restriction of salt radial translocation in roots is crucial to control the root-to-shoot salt transport [27,28]. Using selective microelectrodes, the Na^+^ flux of the root xylem was measured in the salt-stressed poplars, as the Na^+^ flow in the root xylem can reflect the real-time radial transport of Na^+^ in roots. After a short-term salt exposure, the xylem Na^+^ efflux of the two poplars drastically increased with a 3.1–3.5-fold higher flux rate in the *PtEXPA6*-overexpressing lines (Figure 11). Under the no-salt control conditions, the Na^+^ flux in the root xylem of 84K and 717-1B4 was very low or undetectable, and there is no significant difference between the WT and transgenic lines (Figure 11). The greater Na^+^ efflux in the xylem indicates that the transgenic poplars exhibited a lower capacity to restrict the radial translocation of Na^+^ salt to the xylem under saline conditions. Noteworthily, we observed that the PtEXPA6-promoted CW loosening facilitated the radial translocation of Na^+^ in roots. The PtEXPA6-promoted radial translocation of Na^+^ in roots was markedly restricted by the addition of 300 mOsmol kg^−1^ mannitol (osmotic potential is −0.75 MPa, Figure 11), which induces contraction and hardens the cell walls (Figure 10) [29,30]. We noticed that the Na^+^ flux of the xylem in the contracted roots was resumed after an 0.1 MPa osmotic jump (Figure 11), which induces the extension of the cell walls (Figure 10) [29,30]. This Na^+^ kinetics upon an osmotic jump demonstrated that CW loosening favors the radial Na^+^ transport, while a CW contraction restricts the Na^+^ transport in poplar roots.

### 2.10. Na^+^ Flux of Leaf Petiole Vascular Bundles and the Response to Osmotic Jump

**Figure 12:**
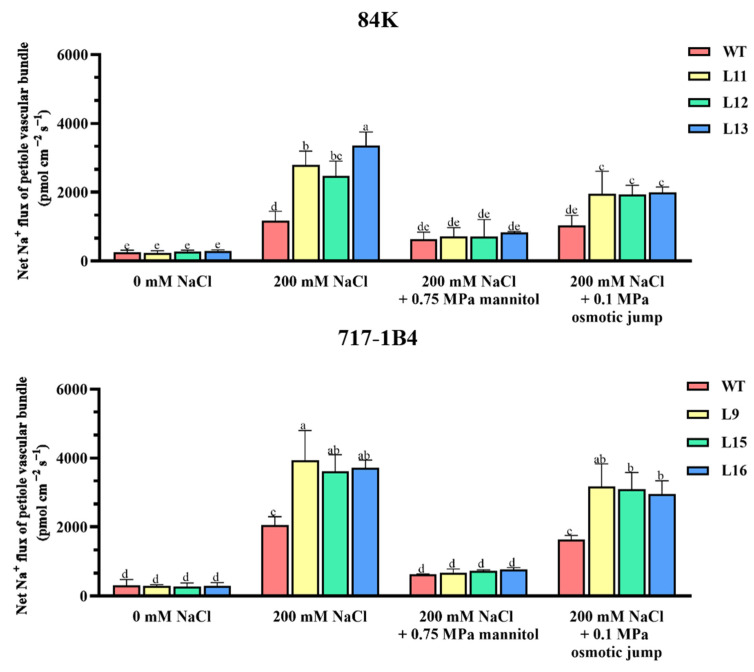
Na^+^ flux of the petiole vascular bundle and the response to an osmotic jump in the wild-type and *PtEXPA6*-overexpressing lines of 84K and 717-1B4. After exposure to 200 mM NaCl for 4 h, the intact root tips of the *PtEXPA6*-trangenic lines of 84K (L11, L12, and L13) and 717-1B4 (L9, L15, and L16), and wild-type (WT) were exposed to 300 mOsmol kg^−1^ mannitol (−0.75 MPa), followed by a 0.1 MPa osmotic jump. The net Na^+^ flux of the petiole vascular bundle was measured before and after the addition of mannitol, and the subsequent 0.1 MPa osmotic jump. The data are means ± SD (*n* = 3), and the bars with different letters indicate significant differences (*p* < 0.05).

The radial translocation of Na^+^ salt to the xylem would result in a higher rate of salt transport to the shoots. Thus, the Na^+^ flux of the vascular bundles in the leaf petiole was examined to determine the real-time Na^+^ translocation kinetics from the roots to the leaves [31]. The roots of intact plants were subjected to short-term salt exposure, the leaf petiole was immobilized in a measuring chamber after the leaf blade was removed. The Na^+^ flux of the vascular bundles in the leaf petiole was immediately measured with selective microelectrodes. The xylem Na^+^ efflux of the vascular bundles drastically increased in the two salinized poplars with a 1.9–2.9-fold higher flux rate in the *PtEXPA6*-overexpressing lines (Figure 12). Under no-salt control conditions, the Na^+^ flux in the leaf vascular bundles of 84K and 717-1B4 was very low or undetectable, and there is no significant difference between the WT and transgenic lines. We also found that the *PtEXPA6*-promoted cell wall loosening facilitated the root-to-shoot Na^+^ transport. The Na^+^ flux of the vascular bundles in the leaf petiole were restricted by 300 mOsmol kg^−1^ mannitol (−0.75 MPa), while the Na^+^ flux was markedly recovered upon an 0.1 MPa osmotic jump (Figure 12), which is similar to the Na^+^ flux of the root xylem (Figure 11). The real-time Na^+^ translocation kinetics upon an osmotic jump demonstrated that CW loosening favors the Na^+^ transport from the roots to the leaves, while a CW contraction restricts the root-to-shoot Na^+^ transport.

### 2.11. The Correlation Analysis and Principal Component Analysis (PCA)

The correlation analysis and principal component analysis (PCA) of the measured traits showed that there are species differences in the correlations among the characteristics and principal components (Tables S3–S6). The correlation analysis showed significant positive and negative correlations among the growth, physiological, and biochemical measurements, in particular the clone 717-1B4. For example, stem growth, gas exchange, chlorophyll fluorescence, activity, and the transcription of antioxidant enzymes had high positive correlations (ca. 0.800–0.999), with a few exceptions (e.g., CAT activity and *SOD [Cu-Zn]* expression). Significant negative correlations were also noted between Na^+^ content, Na^+^ fluxes, relative electrolyte leakage, and salt-resistant characteristics, such as stem growth, gas exchange, chlorophyll fluorescence, activity, and the transcription of antioxidant enzymes (Table S3). Compared with the clone 717-1B4, the 84K poplar showed relatively lower correlations among the measured characteristics. In general, leaf gas exchange and chlorophyll fluorescence had low correlations with the antioxidant enzymes, Na^+^ content and fluxes, and relative electrolyte leakage, although there are significant positive correlations between gas exchange, chlorophyll fluorescence, and stem height growth (Table S4). For the 84K poplar, significant negative correlations were always observed between the Na^+^ content, Na^+^ fluxes, and antioxidant enzymes, which is similar to the clone 717-1B4 (Tables S3 and S4).

The PCA revealed variations among the traits and determined three main factors that explained almost 100% of the total variance for the two hybrid poplars, 717-1B4 and 84K (Tables S5 and S6). It is shown that the three PCs displayed eigenvalues higher than significant although the eigenvalues varied within species (Tables S5 and S6). For the clone 717-1B4, the first principal component (PC1), second principal component (PC2), and third principal component (PC3) accounted for 84.737%, 8.120%, and 7.144% of the observed variations, respectively (Table S5). For the 84K poplar, PC1, PC2, and PC3 accounted for 62.782%, 26.080%, and 11.138% of the observed variations, respectively (Table S6). There are also species differences in the traits included in PC1 and PC2. For clone 717-1B4, the PC1 covered the stem growth, gas exchange, chlorophyll fluorescence, activity and transcription of antioxidant enzymes, and Na^+^ content and fluxes (Table S5). But the gas exchange, i.e., net photosynthetic rate (Pn), transpiration rate (E), and stomatal conductance (Cleaf), fell into PC2 for the 84K poplar (Table S6). The PC2 also contained traits like relative electrolyte leakage, SOD activity, and Na^+^ flux of petiole at −0.75 MPa mannitol (Table S6).

## 3. Discussion

### 3.1. PtEXPA6 Negatively Regulates Salt Tolerance in Transgenic Poplars

The *PtEXPA6* transgenic lines of 84K and 717-1B4 showed a more pronounced reduction in stem height and diameter growth compared to the WT poplars after 15 days of exposure to 100 mM NaCl (Figure 4). The salt-reduced growth was associated with a drastic reduction in the photosynthetic capacity of the transgenic poplars (Figure 5 and Figure 6), as there are significant positive correlations between gas exchange, chlorophyll fluorescence, and stem growth for the two poplars (Tables S3 and S4). Thus, *PtEXPA6* increases the salt sensitivity of the transgenic poplars of both 84K and 717-1B4. This is consistent with the Arabidopsis expansin genes, *AtEXP3* and *AtEXPβ1*, which resulted in increased sensitivity to salt stress [21]. However, these results are inconsistent with the finding that the overexpression of *NtEXPA4* [10], *AstEXPA1* [11], *AtEXP2* [12], *SmEXPA23* [13], *PttEXPA8* [14], and *CqEXPA50* [20] increased the salt tolerance of the transgenic plants. These contrasting results suggest that expansin proteins play different roles in regulating plant responses to salinity. The positive and negative effects of expansins in salt tolerance are probably related to the function of expansin in mediating water and ionic relations that are associated with the expansin-modified morphological structures. RhEXPA4 leads to smaller, compact cells, fewer stomata, and vascular patterning in the center of the leaf epidermis [16]. This suggests that RhEXPA4 might confer salt tolerance by reducing water loss. In contrast, the size of the leaves in *AtEXP3-* and *AtEXPβ1*-overexpressed plants was larger than those of the wild type [21]. The increased transpiration surface would lead to greater water consumption under salt stress, resulting in an increased salt sensitivity in *AtEXP3*- and *AtEXPβ1*-overexpressed plants. There are increasing evidence showing that expansins confer salt tolerance by improving primary root length and root architecture. RhEXPA4, OsEXPA7, NtEXPA11, and TaEXPB23 have been shown to enhance the root growth and overall development of the root system [15,16,17,18]. This could enhance the uptake of more water and nutrients, resulting in better plant growth and development under salt stress. Moreover, wheat TaEXPA2 and rice OsEXPA7 have been shown to reduce Na^+^ in leaves and roots under salt stress [17,19]. It is hypothesized that the longer metaxylem cells in *OsEXPA7*-overexpressed plants can take up more water than the WT plants, reducing the Na^+^ concentration both in the cytoplasm and in the vacuoles [17]. However, our results show that the overexpression of *PtEXPA6* leads to a loosening of the cell walls but does not promote root growth under long-term salt stress. At present, it is unknown whether PtEXPA6 affects the length of the metaxylem cells in poplar roots. Therefore, the PtEXPA6-modified cell wall loosening and morphological structures and the significance for water and Na^+^ transport need to be further investigated.

The pattern of *PtEXPA6* transcription contrasts with that of salt-induced expansins in wheat leaves and roots, such as *TaEXPB2-A*, *TaEXPA3-A*, *TaEXPB4-A*, *TaEXPA6-A*, *TaEXPA9-A*, and *TaEXPB10-A* (Figure 1) [9]. Thus, the downregulation of *PtEXPA6* favors the adaptation of *Populus trichocarpa* to saline conditions (Figure 1). The suppression of salt tolerance by *PtEXPA6* was mainly due to excessive Na^+^ accumulation, which resulted in a reduced ability to maintain photosynthesis and ROS homeostasis in the transgenic poplars (Figure 5, Figure 6, Figure 7 and Figure 8). 

### 3.2. PtEXPA6 Increases Na^+^ Transport from Root to Shoot under Salt Stress

The *PtEXPA6*-overexpressing lines of 84K and 717-1B4 showed greater Na^+^ accumulation in roots, stems, and leaves compared to the WT poplars (Figure 9). The accumulation of Na^+^ ions was due to the increased radial salt translocation in the roots and the subsequent salt transport from the root to the shoot, as the Na^+^ content was positively correlated to the Na^+^ fluxes in roots and leaf petiole (Tables S3 and S4). The flux data showed that the Na^+^ efflux of the root xylem increased dramatically in the *PtEXPA6*-overexpressing lines of both poplars (Figure 11). This indicates that the transgenic poplars had a reduced ability to limit the radial translocation of Na^+^ into the xylem under salt stress. The increased radial translocation of Na^+^ salt into the xylem would lead to a higher rate of salt transport to the shoots, as the Na^+^ flux of the vascular bundles in the petiole increased significantly under salt conditions (Figure 12). The *PtEXPA6*-stimulated Na^+^ translocation in roots and shoots was probably due to the CW loosening, which facilitated Na^+^ transport in the apoplast. In accordance, the overexpression of *PtEXPA6* increased the CW extensibility in poplar roots (Figure 10). The CW loosening would lead to an increase in CW volume and hydraulic conductivity, stimulating the flow of solution with high Na^+^ concentration. This probably increases the radial translocation of Na^+^ salt into the xylem and the subsequent salt transport to the shoots. The apoplastic pathway has been shown to be responsible for up to 50% of the total Na^+^ and Cl^−^ uptake by the root [32,33]. It is noteworthy that the *PtEXPA6*-promoted radial translocation of Na^+^ in roots and root-to-shoot transport were both restricted by the application of 300 mOsmol kg^−1^ mannitol (Figure 11 and Figure 12), which induces the contraction and hardening of the cell walls (Figure 10) [29,30]. Furthermore, the Na^+^ transport was resumed when the contracted roots were exposed to a 0.1 MPa osmotic jump (Figure 11 and Figure 12), which induces the extension of the cell walls (Figure 10). Collectively, this transient Na^+^ kinetics in response to a 0.1 MPa osmotic jump demonstrated that CW loosening favors the apoplastic Na^+^ transport, while a CW contraction restricts the radial translocation of Na^+^ in roots and root-to-shoot transport. The wild-type 84K and 717-1B4 showed a lower contractibility and extensibility than the transgenic lines, this could benefit the salt-stressed poplars to restrict the radial and longitudinal transport of sodium ions (Figure 10, Figure 11 and Figure 12). Consistent with this, the long-term salt-stimulated CW stiffening, observed at high solution ionic strength, contributes to the decrease in CW swelling capacity [34]. The resulting decrease in CW volume and hydraulic conductivity restricts the flow of solution with high Na^+^ concentration. This probably allows the root cells to adapt to the stress conditions and prevents Na^+^ and Cl^−^ from entering the xylem [35]. In our study, the Na^+^ transport stimulated by *PtEXPA6* contradicts the findings that wheat *TaEXPA2* and rice *OsEXPA7* lead to a reduction in Na^+^ in the leaves and roots under salt stress [17,19]. These contrasting results suggest that expansin proteins play different roles in regulating the ionic relations and salt tolerance of salinized plants. It has been suggested that expansins confer this ability to remodel cell wall composition and maintain cell wall flexibility in roots under NaCl stress, contributing to improved root architecture and salt tolerance [16]. In the *OsEXPA7*-OX rice plants, the longer metaxylem cells in the primary roots showed that cell elongation occurs through expansin-mediated cell wall loosening [17]. Similarly, the transgenic *RhEXPA4* plants had longer primary roots and more lateral roots under salt stress [16]. It is assumed that the longer metaxylem cells can take up more water than the WT plants, thus reducing Na^+^ concentration in both the cytoplasm and vacuoles [17]. However, our results show that the overexpression of *PtEXPA6* leads to a loosening of the cell wall, which facilitates the radial translocation of Na^+^ into the root xylem and the subsequent Na^+^ translocation from the roots into the leaves (Figure 10, Figure 11 and Figure 12). The correlation analysis revealed that in the two poplars, particularly 717-1B4, Na^+^ content and flux showed a positive correlation to relative electrolyte leakage but are usually negatively correlated with stem growth, gas exchange, chlorophyll fluorescence, and activity and transcription of antioxidant enzymes (Tables S3 and S4). Therefore, the downregulation of *PtEXPA6* would result in the restriction of Na^+^ transport and favor *Populus trichocarpa* in maintaining growth, photosynthesis, and ROS homeostasis under saline conditions (Figure 4, Figure 5, Figure 6, Figure 7 and Figure 8).

### 3.3. PtEXPA6 Influences Photosynthesis under Salt Stress

The long-term salinity resulted in a more pronounced reduction in Pn in the transgenic lines of 84K (L11, L12, and L13) and 717-1B4 (L9, L15, and L16) compared to the WT (Figure 5). The salt-decreased photosynthetic capacity partly resulted from the decrease in stomatal conductance as a positive correlation was observed between Pn and stomatal conductance (Figure 5, Tables S3 and S4). Additionally, the salt-reduced activity of the PSII reaction center and decrease in ETR might also affect the photosynthetic process in the *PtEXPA6*-transgenic poplars as Pn positively correlated with chlorophyll fluorescence (Figure 6, Tables S3 and S4). We have previously shown that the excessive accumulation of salt ions leads to decreased stomatal conductance and photosynthetic capacity [36,37]. It is possible that PtEXPA6 enhanced salt transport from roots to shoots, and the great buildup of Na^+^ in the chloroplasts of the transgenic poplars of 84K and 717-1B4 exhibit direct and indirect restrictions on dark and light reactions, as Na^+^ content and fluxes are negatively correlated with gas exchange and chlorophyll fluorescence, in particular clone 717-1B4 (Figure 9, Tables S3 and S4). 

### 3.4. PtEXPA6 Influences ROS Scavenging Capacity under Salt Stress

NaCl caused significant activation of antioxidant enzymes, POD, SOD, and CAT, in the WT and transgenic poplars (Figure 7). This was mainly due to the salt-increased transcripts of *CAT1* and *PODa2* (Figure 8), as the enzymic activity was positively correlated with the transcript abundance of the antioxidant enzymes (Tables S3 and S4). However, in the salt-stressed poplars, the activity and transcription of the antioxidant enzymes were typically lower in the *PtEXPA6*-overexpressing lines (Figure 7 and Figure 8). The less stimulated activity and transcription of antioxidant enzymes resulted in the inability to efficiently remove salt-induced ROS during long-term salinity. In accordance, REL was significantly higher in the transgenic 84K and 717-1B4 than in the WT poplars (Figure 7). Our results are consistent with the finding that the overexpression of wheat *TaEXPA2* or *Chenopodium quinoa CqEXPA50* improves the salt tolerance of transgenic plants by enhancing the enzymatic antioxidant system [19,20]. Apparently, the lower activation of the antioxidant enzymes in the *PtEXPA6*-overexpressing lines was at least partly the result of the excessive accumulation of salt ions, which led to increased ROS production and decreased ROS scavenging capacity [36,37]. Here, Na^+^ content and fluxes also showed a negative correlation with the activity and transcription of antioxidant enzymes (Tables S3 and S4). Consistent with this, we previously found that salt exposure in *P. popularis* leaves increased the activity of ascorbate peroxidase (APX), CAT, and glutathione reductase (GR) [36,37]. However, the salt-produced ROS exceeded the antioxidant capacity of the enzymatic system, leading to oxidative damage in the salt-sensitive poplars [36,37].

## 4. Materials and Methods

### 4.1. Total RNA Isolation, PtEXPA6 Cloning, and Sequence Analysis

Total RNA was isolated from the leaves of *Populus trichocarpa* using the E.Z.N.A.^TM^ Plant RNA Kit (Omega Bio-Tek, Guangzhou, China). The upper leaves (3rd to 8th from the top) were collected from the soil-cultured plants. The removal of genomic DNA and the synthesis of the first strand of cDNA were performed using the reverse transcriptase kit HiFiScript gDNA Removal RT Master Mix (CoWin Biotech, Taizhou, China). *PtEXPA6* was cloned by PCR amplification and the reaction mixture (50 µL) contained 2 µL cDNA product, 25 µL KOD One™ PCR Master Mix (TOYOBO, OSAKA, JAPAN) and 1 µL specific primers (10 µM), 5′-ATGGCAATGAGCAGTTTAA-3′ (forward), and 5′-GACCCTGAAATTCTTGCCGG-3′ (reverse). The multiple sequence alignments of the EXPA proteins were performed using ClustalW (http://www.genome.jp/tools/clustalw/, EMBL-EBI, Hinxton, Cambridgeshire, UK, accessed on 18 August 2020). The phylogenetic tree was created using the software MEGA11 (http://www.megasoftware.net, the Centre for Evolutionary Medicine and Informatics, Tempe, AZ, USA, accessed on 16 February 2023). The accession numbers of the expansin orthologs used for the multiple sequence alignment and phylogenetic analysis are listed in Supplementary Table S1.

### 4.2. PtEXPA6 Overexpression in Poplars

The coding sequence of *PtEXPA6* was ligated into the pART-CAM-FLAG vector, in which the Xho I and Xba I sites were driven by the cauliflower mosaic virus (*CaMV*) *35S* promoter. Subsequently, the *PtEXPA6* overexpression construct was transferred into *Agrobacterium tumefaciens* (strain GV3101) for plant transformation. A total of 10 transgenic lines of *Populus alba* × *P. tremula* var. *glandulosa* (84K) and 16 lines of *Populus tremula* × *P. alba* INRA ‘717-1B4’ were obtained and used for PCR detection. Five transgenic lines of 84K and six transgenic lines of 717-1B4 were confirmed with a Western blot analysis. The 1/2MS solid medium was used to propagate regenerated plantlets, and the medium was supplemented with 6 g of agar, 30 g of sucrose, 0.05 mg/L of IBA, and 0.02 mg/L of NAA, and pH was adjusted to 5.8. The light intensity in the tissue culture room was 100–200 μmol m^−2^s^−1^ with the photoperiod being 16 h light/8 h dark. The relative humidity was 75%, and the temperature was 22 ± 1 °C. Poplar plantlets (5–10 cm high) were transplanted into individual pots (1000 mL) for soil culture. The matrix was a mixture of peat soil and quartz sand (peat soil/quartz sand = 1:1). The soil culture conditions were the same as those in the culture room. 

### 4.3. Western Blotting

The Western blotting of PtEXPA6 was performed as previously described [38]. In brief, total protein was isolated from the leaf samples with an extraction buffer (50 mM Tris-MES, pH 7.5, 80 mM NaCl, 10 mM MgCl_2_, 10% glycerol, 0.2% NP-40, 1 mM EDTA, 1 mM PMSF, and protease inhibitor cocktail [Roche (China) Holding Ltd. Shanghai, China]). Sodium dodecyl sulfate-polyacrylamide gel electrophoresis (SDS-PAGE) and the subsequent immunoblotting were performed according to standard procedures. PtEXPA6 protein immunoblots were performed using mouse monoclonal anti-FLAG antibody (Abclonal, Wuhan, China, cat no: AE005) at 1:5000 dilution. Actin (26F7) mAb for PLANTs (Abmart, Shanghai, China, cat no: M20009L) was used to detect endogenous actin protein, which served as a loading control. Secondary HRP-conjugated Goat anti-Mouse IgG (H + L) (Abclonal, Wuhan, China, cat no: AS003) was used at 1:5000 for detection via the eECL Western Blot Kit (CoWin Biosciences, Beijing, China, cat no: CW0049S). Protein gel blots were imaged by the ChemiDoc MP system (Bio-Rad Laboratories, Inc., Hercules, CA, USA). The Western blotting was repeated three times and similar results were obtained.

### 4.4. Comparative Extensibility and Contractility of Root Tips

To determine the loosening of the cell wall promoted by *PtEXPA6*, the comparative extensibility and contractility of intact root tip tissue were measured in wild-type and transgenic poplars [25,26]. The comparative contractility was determined by assessing the initial root contraction elongation rate (1 min) induced by 300 mOsmol kg^−1^ mannitol (−0.75 MPa). The comparative elongation capacity of the growing tip tissue was then directly determined by an osmotic jump method [25,26]. Briefly, the wild-type and *PtEXPA6*-overexpressing lines of 84K (L11, L12, and L13) and 717-1B4 (L9, L15, and L16) were grown hydroponically in 1/2 MS culture medium for one week. The primary root of an intact plant was fixed on a Petri dish before the addition of 300 mM mannitol (−0.75 MPa). Osmotically induced changes in the position of the growing tip began within 20 s of the addition of mannitol, and the determination of the initial rate of mannitol-induced contraction was completed after 20–30 s to minimize deviation from initial conditions. The comparative contractility is the difference between the root apex length and the initial root apex length per unit time after mannitol treatment and is expressed in µm min^−1^ 0.75 MPa^−1^. Comparative extensibility was then determined by assessing the 1 min initial increase in root elongation rate induced by an osmotic jump of 0.1 MPa [25]. The comparative elongation rate is the difference between the length of the apical site per unit time of elongation and the length of the initial apical site, expressed in µm min^−1^ 0.1 MPa^−1^. Changes in the root tip position were observed using an ML31 biomicroscope (Mshot, Guangzhou, China) and determined using an Mshot Image Analysis System (Mshot, Guangzhou, China). Three root tips were measured for each plant, and three independent individuals were biologically replicated for each treatment.

### 4.5. Phenotype Test of Salt Tolerance

#### 4.5.1. Measurement of Growth

Uniform plants of the wild-type (WT) and *PtEXPA6*-overexpressing lines of 84K (L11, L12, and L13) and 717-1B4 (L9, L15, and L16) were treated with NaCl saline (0 or 100 mM) for 15 days. The light intensity in the tissue culture room was 100–200 μmol m^−2^s^−1^ with the photoperiod being 16 h light/8 h dark. The relative humidity was 75%, and the temperature was 22 ± 1 °C. Shoot height and stem diameter were measured at the end of the experiment. The relative growth of the stem height and diameter during the observation period was calculated as folllows: (H1−H0)/H0 or (D1−D0)/D0. H1 (cm) and H0 (cm) are the stem height at the end and beginning of the salt treatment. D1 (mm) and D0 (mm) are the stem diameter at the end and beginning of the salt treatment. For the WT and transgenic lines of two poplars, six individual plants were established for the control and salt treatment. 

#### 4.5.2. Measurement of the Relative Electrolyte Leakage (REL)

The upper leaves of the shoot (3rd to 8th from the top) were sampled from the WT and *PtEXPA6*-overexpressing lines of 84K (L11, L12, and L13) and 717-1B4 (L9, L15, and L16) after 15 days of NaCl treatment (0 or 100 mM). The REL was calculated from the initial relative conductivity (EC1) before boiling and the final conductivity (EC2) after boiling: REL (%) = (EC1/EC2) × 100%. Three to four individual plants of each genotype were used for each treatment.

#### 4.5.3. Measurement of Leaf Gas Exchange and Chlorophyll Fluorescence

Leaf gas exchange and chlorophyll fluorescence were measured after the WT poplar and the *PtEXPA6*-overexpressing lines of 84K (L11, L12, and L13) and 717-1B4 (L9, L15, and L16) were treated with NaCl (0 or 100 mM) for 15 days. The net photosynthetic rate (Pn, μmol m^−2^s^−1^), transpiration rate (E, mmol m^−2^s^−1^), and stomatal conductance (Cleaf, mmol m^−2^s^−1^) of the upper mature leaves (6th–8th from the top) were measured using a portable open gas exchange system, the LI-6400 (Li-Cor, Inc., Lincoln, NE, USA). The gas exchange was measured with light intensity at 150 μmol m^−2^s^−1^, relative humidity 75%, and temperature was 22 ± 1 °C. The maximum photochemical efficiency of PSII (Fv/Fm), the actual photosynthetic quantum yield (YII), and the relative electron transport rate (ETR) were analyzed with a pulse-amplitude-modulated (PAM) chlorophyll fluorometer, the JUNIOR-PAM (HeinzWalz GmbH, Effeltrich, Germany) [39]. The leaves were dark-adapted for 20 min, and fluorescence was measured at 22 ± 1 °C.

### 4.6. Determination of Antioxidant Enzyme Activity

The WT and PtEXPA6-overexpressing lines of 84K (L11, L12, and L13) and 717-1B4 (L9, L15, and L16) were salinized with NaCl (0 or 100 mM) for 15 days. Samples were taken from the leaves (3rd to 8th from the top) and used to measure the total activity of antioxidant enzymes. POD, SOD, and CAT activity (U g^−1^) was analyzed using assay kits for POD (BC0090), CAT (BC0205), and SOD (BC0175), respectively (Beijing Solarbio Science & Technology, Beijing, China) [39]. One unit of SOD was defined as the amount of enzyme causing 50% inhibition of the reaction compared with a blank sample. The unit of CAT activity was defined as the consumption of 1 μmol H_2_O_2_ per minute per gram of plant tissue. One unit of enzyme activity of POD was defined as the change in absorbance at 470 nm by 0.01 in a milliliter of the reaction system per minute per gram of tissue. 

### 4.7. Na^+^ Concentration in the Roots, Leaves and Stems

Roots, stems, and leaves were collected from the soil-cultured WT and *PtEXPA6*-overexpressing lines of 84K (L11, L12, and L13) and 717-1B4 (L9, L15, and L16) after 15 days of salt treatment (0 or 100 mM NaCl). The oven-dried samples (60 °C, 5 days) were digested with H_2_SO_4_-H_2_O_2_ and used for the Na^+^ content (mmol g^−1^ DW) determination with an atomic absorption spectrometer (Varian SpectrAA 220FS, Palo Alto, CA, USA) [39]. Three to four individual plants of each genotype were used for each treatment.

### 4.8. Flux Records of Na^+^ in the Root Xylem and Leaf Petiole Vascular Bundle

The net Na^+^ flux (pmol cm^−2^s^−1^) in the root xylem was recorded using a non-invasive micro-test system (NMT-YG-100, Younger USA, LLC, Amherst, MA, USA) [40]. After short-term salt exposure (200 mM, 4 h), the roots with tips and mature zones of approximately 10–15 cm in length were collected from the control and salt-stressed poplars of WT and *PtEXPA6*-overexpressing lines 84K (L11, L12, and L13) and 717-1B4 (L9, L15, and L16). The roots were equilibrated for 30 min in a measuring solution (0.1 mM NaCl, 0.1 mM CaCl_2_, 0.1 mM MgCl_2_, and 0.5 mM KCl, pH 5.7). The selective microelectrodes for Na^+^ were calculated and used to monitor the net flux of Na^+^ in the xylem of the maturation zone. Continuous recordings were made at each measurement point for 5–8 min and the average flux was calculated. Three to four individual plants of each genotype were used for flux recording. The response of the root xylem Na^+^ flux to osmotic jump was also examined in WT and transgenic poplars. After the NaCl treatment as described above, 300 mOsmol kg^−1^ mannitol (−0.75 MPa) was added to a measuring solution, and real-time Na^+^ flux was recorded in the root xylem. Thereafter, the concentration of mannitol in the measuring solution was routinely diluted with an 0.10 MPa increase in external osmotic potential, and Na^+^ flux kinetics was immediately measured in the root xylem.

Real-time translocation kinetics of Na^+^ flux from vascular bundles in the petiole was performed as described previously [31]. In brief, the entire root system of intact plants was exposed to a short-term salt stress (200 mM NaCl, 4 h). Then, the petiole was immobilised in the measurement chamber after the leaf blade was removed. The Na^+^ flux of the vascular bundles in the petiole was immediately measured with NMT microelectrodes as described above. The response of the Na^+^ flux in the leaf petiole vascular bundles to osmotic jump was also examined in the WT and transgenic poplars. After 300 mOsmol kg^−1^ mannitol was added to the NaCl-treated roots, Na^+^ flux kinetics was observed in the leaf petiole vascular bundle. Thereafter, the concentration of mannitol was diluted with an 0.10 MPa increase in external osmotic potential, and the Na^+^ flux kinetics was immediately measured in the leaf petiole vascular bundles. Three to four individual plants of each genotype were used for the flux recording of the petiole vascular bundles.

### 4.9. RT-qPCR Analysis

*P. trichocarpa* was subjected to treatment with 0 or 100 mM NaCl for 48 h. The roots, stem, and mature leaves in the upper shoot were collected after 0, 3, 6, 12, 24, and 48 h of NaCl treatment and used for the RT-qPCR analysis of *PtEXPA6*. The transcripts of *PODa2*, *SOD [Cu-Zn]*, and *CAT1* in the WT and transgenic poplars were analyzed under the control and NaCl treatments (100 mM, 15 days). RNA isolation from *P. trichocarpa*, 84K, and 717-1B4 was performed using the E.Z.N.A.^TM^ Plant RNA Kit (Omega Bio-Tek, Guangzhou, China). Subsequently, the RNA (1 µg) was used for reverse transcription with Moloney murine leukemia virus (M-MLV) reverse transcriptase and an oligo (dT) primer (Promega, Madison, WI, USA) according to the manufacturer’s recommended protocol. RT-qPCR was performed using the LineGene 9600 Plus (Bioer Technology, Hangzhou, China) and *UBQ* served as an internal control for *P. trichocarpa*, 84K and 717-1B4 [41]. Three individual biological replicates were generated for each treatment.

### 4.10. Data Analysis

Na^+^ fluxes were calculated using JCal V3.2.1, a free MS Excel spreadsheet developed by Yue Xu (http://www.xuyue.net/, accessed on 5 May 2024). All the experimental data were statistically analyzed using SPSS version 19.0 (IBM Corporation, Armonk, NY, USA). The correlation analysis and principal component analysis (PCA) were also performed using the SPSS software (version 19.0). The one-way ANOVA method was used to compare mean values between the treatments. *p* < 0.05 or *p* < 0.01 was considered a significant difference unless otherwise stated.

## 5. Conclusions

In summary, the overexpression of *PtEXPA6* reduces plant growth, photosynthetic capacity, and ROS scavenging capacity under salt stress, which is due to the excessive Na^+^ accumulation in roots and shoots. The *PtEXPA6*-transgenic poplars exhibited a more pronounced increase in the radial translocation of Na^+^ salt into the root xylem and the translocation of Na^+^ from roots to leaves under salt stress. Moreover, PtEXPA6 increased the root contractability and extensibility in transgenic poplars. Therefore, we hypothesize that the overexpression of *PtEXPA6* results in cell wall loosening, which leads to an increase in CW volume and hydraulic conductivity, stimulating the flow of solution with high Na^+^ concentration. As a result, the radial translocation of Na^+^ into the root xylem and the subsequent Na^+^ translocation from roots to leaves were consequently increased under salt stress, resulting in excessive Na^+^ accumulation and reduced salt tolerance. Therefore, the downregulation of *PtEXPA6* in the NaCl-treated *Populus trichocarpa* would lead to a restriction of Na^+^ accumulation, thus favoring the maintenance of photosynthesis and ROS homeostasis under saline conditions.

## Data Availability

The original contributions presented in the study are included in the article/Supplementary Material, further inquiries can be directed to the corresponding author.

## Supplementary Materials

The following supporting information can be downloaded at: https://www.mdpi.com/article/10.3390/ijms25179354/s1.
