# Supplementary material for: Populus trichocarpa EXPA6 Facilitates Radial and Longitudinal Transport of Na+ under Salt Stress
Source: Int J Mol Sci. 2024 Aug 29;25(17):9354. doi: 10.3390/ijms25179354 (PMC11395417; doi:10.3390/ijms25179354)
Supplement: Supplementary file 1 [file ijms-25-09354-s001.zip › ijms-3175313-supplementary.pdf]

International Journal of
Molecular Sciences

MDPI

Supplementary Table S1. Primers used for quantitative real-time PCR.

Gene	Forward primer	Reverse prime
PtUBQ	AGACCTACACCAAGCCCAAGAAGAT	CCAGCACCGCACTCAGCATTAG
PtEXPA6	TGACGCACAATCCCACTATC	GTAGGCAACAGGGACGATAC
PODa1	CCCGATGCCTTTGACAGTAAC	GAGGGCTGAGATTTCCCATTC
SOD[Cu-Zn]	GTTGGTGATGATGGCACTGCTACTT	ACCGGTGGTTTTGCTGAGTTCATGT
CAT1	AGTAGGAGGCACAAATCACAG	TAAACACCAGGAACCACAATA

Supplementary Table S2. Accession numbers of EXPA orthologs.

	Accession number
PtEXPA6	XP_002298353.1
PtEXPA1	XP_006378591.1
PeEXPA6	XP_011035307.1
PeEXPA1	XP_011017384.1
AtEXPA6	NP_180461.1
AtEXPA1	NP_177112.1
NtEXPA6	XP_016500095.1
NtEXPA1	XP_016452402.1
NsEXPA6	XP_009761115.1
NsEXPA1	XP_009789308.1
GmEXPA6	XP_003544219.1
GmEXPA1	XP_040867070.1
ZmEXPA6	ACG39445.1
ZmEXPA1	NP_001105040.1
OsEXPA6	NP_001404595.1
OsEXPA1	NP_001406039.1
PttEXPA1	AAR09168.1
PttEXPA2	AAR09169.1
PaEXPA6	XP_034887419.1
PaEXPA1	XP_034906718.1
SvEXPA4	KAJ6720454.1
CmEXPA6	QDL52549.1
CmEXPA1	XP_050937041.1
RrEXPA6	XP_062015166.1
RrEXPA1	XP_062014857.1
ZjEXPA6	XP_015874026.2
ZjEXPA1	XP_015883469.3
GaEXPA6	XP_017610980.1
GaEXPA1	XP_017637923.1
PvEXPA6	XP_031273596.1
PvEXPA1	XP_031281765.1
MnEXPA6	XP_010106493.1
MnEXPA1	XP_010093601.2
PpEXPA6	XP_007205740.1
PpEXPA1	XP_007209512.1
HsEXPA6	XP_039047640.1
HsEXPA1	KAE8733953.1
SoEXPA6	XP_030464354.1
SoEXPA1	XP_030470632.1
PtEXPA4	XP_002313434.1
PtEXLB1	XP_006385603.1
PaEXLB1	XP_034932610.1
GmEXLB1	XP_003517382.1
PtEXPB3	XP_002319359.2
AtEXPB1	NP_179668.1
PeEXPB3	XP_011039743.1
PaEXPB3	TKR90618.1
SpEXPB3	KAJ6744980.1
PtEXLA2	XP_002313682.3

PeEXLA2	XP_011045305.1
SvEXLA1	XP_011045305.1
PaEXLA2	XP_034927552.1

International Journal of *Molecular Sciences*

MDPI

Supplementary Table S3. Correlation analysis of measured traits of wild type and *PtEXPA6*-transgenic poplars of 717-1B4.

	Height growth	Diameter growth	Pn	E	Cleaf	ETR	Y(Ⅱ)	Fv/Fm	POD activity	SOD activity	CAT activity	Relative electrolyte leakage	*PODa2* relative expression level	*SOD [Cu-Zn]* relative expression level	*CAT1* relative expression level	Na^+ Content of Root	Na^+ Content of Stem	Na^+ Content of Leaf	Na^+ flux of root (200mM NaCl)	Na^+ flux of root (0.75 MPa Mannitol)	Na^+ flux of root (0.1 MPa Mannitol)	Na^+ flux of petiole (200mM NaCl)	Na^+ flux of petiole (0.75 MPa Mannitol)	Na^+ flux of petiole (0.1 MPa Mannitol)
**Height growth**	1																							
**Diameter growth**	0.970**	1																						
**Pn**	0.937**	0.845*	1																					
**E**	0.993**	0.955**	0.965**	1																				
**Cleaf**	0.908**	0.881**	0.946**	0.948**	1																			
**ETR**	0.999**	0.970**	0.922**	0.986**	0.884**	1																		
**Y(Ⅱ)**	0.998**	0.954**	0.959**	0.997**	0.922**	0.994**	1																	
**Fv/Fm**	0.913**	0.964	0.714**	0.868**	0.725	0.927**	0.883**	1																
**POD activity**	0.985**	0.977**	0.933**	0.991**	0.957**	0.976**	0.983**	0.891**	1															
**SOD activity**	0.951**	0.900**	0.983**	0.980**	0.987**	0.933**	0.965**	0.765	0.972**	1														
**CAT activity**	0.468	0.596	0.434	0.516	0.702	0.435	0.459	0.452	0.612	0.587	1													
**Relative electrolyte leakage**	-0.757	-0.683	-0.906**	-0.827*	-0.946**	-0.722	-0.791	-0.469	-0.819*	-0.922**	-0.637	1												
***PODa2* relative expression level**	0.958**	0.982**	0.882**	0.964**	0.948**	0.947**	0.950**	0.898**	0.990**	0.946**	0.701	-0.794	1											
***SOD [Cu-Zn]* relative expression level**	0.829*	0.939**	0.638	0.807*	0.769	0.828*	0.796	0.927**	0.872**	0.749	0.742	-0.533	0.923**	1										
***CAT1* relative expression level**	0.853*	0.851*	0.890**	0.900**	0.989**	0.825*	0.865**	0.682	0.925**	0.871**	0.795	-0.942**	0.934**	0.781	1									
**Na^+ Content of Root**	-0.944**	-0.857*	-0.999**	-0.971**	-0.952**	-0.929**	-0.964**	-0.728	-0.941**	-0.986**	-0.449	0.905**	-0.893**	-0.656	-0.897**	1								
**Na^+ Content of Stem**	-0.826*	-0.666	-0.942**	-0.850*	-0.788	-0.817*	-0.859**	-0.552	-0.777	-0.863**	-0.119	0.799	-0.685	-0.377	-0.696	0.934**	1							
**Na^+ Content of Leaf**	-0.883**	-0.842*	-0.949**	-0.930**	-0.997**	-0.856**	-0.902**	-0.672	-0.934**	-0.983**	-0.686	0.969**	-0.920**	-0.717	-0.986**	0.953**	0.804*	1						
**Na^+ flux of root (200mM NaCl)**	-0.736	-0.671	-0.885**	-0.809**	-0.942**	-0.699	-0.769	-0.452	-0.807*	-0.909**	-0.674	0.998**	-0.789	-0.538	-0.946**	0.884**	0.763	0.965**	1					
**Na^+ flux of root (0.75 MPa Mannitol)**	-0.830*	-0.704	-0.972**	-0.880**	-0.908**	-0.806*	-0.866**	-0.531	-0.835*	-0.937**	-0.387	0.946**	-0.772	-0.469	-0.857**	0.968**	0.951**	0.929**	0.925**	1				
**Na^+ flux of root (0.1 MPa Mannitol)**	-0.741	-0.655	-0.904**	-0.813*	-0.932**	-0.705	-0.778	-0.438	-0.798	-0.912**	-0.602	0.999**	-0.768	-0.492	-0.924**	0.902**	0.813*	0.958**	0.995**	0.953**	1			
**Na^+ flux of petiole (200mM NaCl)**	-0.982**	-0.956**	-0.960**	-0.996**	-0.970**	-0.971**	-0.987**	-0.854**	-0.996**	-0.988**	-0.590	0.856**	-0.978**	-0.828*	-0.935**	0.966**	0.821*	0.954**	0.843*	0.879**	0.840*	1		
**Na^+ flux of petiole (0.75 MPa Mannitol)**	-0.819*	-0.661	-0.874**	-0.815*	-0.673	-0.824*	-0.842*	-0.613	-0.732	-0.778	0.050	0.632	-0.632	-0.366	-0.559	0.867**	0.967**	0.677	0.588	0.846*	0.646	0.767	1	
**Na^+ flux of petiole (0.1 MPa Mannitol)**	-0.998**	-0.976**	-0.938**	-0.996**	-0.928**	-0.994**	-0.996**	-0.909**	-0.993**	-0.961**	-0.520	0.780	-0.972**	-0.847*	-0.881**	0.946**	0.811*	0.903**	0.761	0.834*	0.762	0.990**	0.791	1

"*" indicates significant correlation ($p < 0.05$); "**" indicates extremely significant correlation ($p < 0.01$).

Supplementary Table S4. Correlation analysis of measured traits of wild type and *PtEXPA6*-transgenic poplars of 84K.

	Height growth	Diameter growth	Pn	E	Cleaf	ETR	Y(Ⅱ)	Fv/Fm	POD activity	SOD activity	CAT activity	Relative electrolyte leakage	*PODa2* relative expression level	*SOD [Cu-Zn]* relative expression level	*CAT1* relative expression level	Na^+ Content of Root	Na^+ Content of Stem	Na^+ Content of Leaf	Na^+ flux of root (200mM NaCl)	Na^+ flux of root (0.75 MPa Mannitol)	Na^+ flux of root (0.1 MPa Mannitol)	Na^+ flux of petiole (200mM NaCl)	Na^+ flux of petiole (0.75 MPa Mannitol)	Na^+ flux of petiole (0.1 MPa Mannitol)
Height growth	1																							
Diameter growth	0.275	1																						
Pn	0.981**	0.109	1																					
E	0.965**	0.115	0.953**	1																				
Cleaf	0.993**	0.178	0.997**	0.960**	1																			
ETR	0.842*	0.319	0.760	0.910**	0.792	1																		
Y(Ⅱ)	0.756	-0.061	0.732	0.898**	0.739	0.926**	1																	
Fv/Fm	0.995**	0.207	0.981**	0.986**	0.990**	0.871**	0.815*	1																
POD activity	0.552	0.826*	0.381	0.519	0.449	0.770	0.492	0.534	1															
SOD activity	-0.055	0.944**	-0.217	-0.219	-0.155	0.018	-0.348	-0.127	0.644	1														
CAT activity	0.669	0.757	0.515	0.646	0.576	0.853**	0.602	0.657	0.988**	0.536	1													
Relative electrolyte leakage	0.179	-0.861**	0.360	0.265	0.289	-0.076	0.242	0.224	-0.683	-0.941**	-0.562	1												
PODa2 relative expression level	0.664	0.710	0.511	0.661	0.571	0.879**	0.649	0.660	0.981**	0.486	0.997**	-0.536	1											
SOD [Cu-Zn] relative expression level	0.850**	0.715	0.766	0.708	0.805*	0.675	0.406	0.800*	0.745	0.460	0.795	-0.278	0.757	1										
CAT1 relative expression level	0.778	0.648	0.646	0.771	0.699	0.928**	0.720	0.775	0.944**	0.386	0.983**	-0.404	0.986**	0.817*	1									
Na^+ Content of Root	-0.727	-0.348	-0.624	-0.819*	-0.663	-0.982**	-0.903**	-0.762	-0.813*	-0.081	-0.875	0.193	-0.907**	-0.586	-0.927**	1								
Na^+ Content of Stem	-0.710	-0.805*	-0.562	-0.651	-0.622	-0.818*	-0.541	-0.685	-0.974**	-0.578	-0.989**	0.554	-0.975**	-0.872**	-0.973**	0.819*	1							
Na^+ Content of Leaf	-0.161	-0.888**	0.032	-0.113	-0.0428	-0.454	-0.143	-0.132	-0.907**	-0.845*	-0.830*	0.923**	-0.818*	-0.502	-0.718	0.553	0.808*	1						
Na^+ flux of root (200mM NaCl)	-0.403	-0.477	-0.252	-0.505	-0.307	-0.816*	-0.691	-0.436	-0.870**	-0.317	-0.866**	0.509	-0.899**	-0.397	-0.849**	0.908**	0.781	0.774	1					
Na^+ flux of root (0.75 MPa Mannitol)	0.387	0.673	0.348	0.132	0.372	0.001	-0.292	0.291	0.303	0.600	0.296	-0.300	0.220	0.739	0.258	0.102	-0.431	-0.259	0.211	1				
Na^+ flux of root (0.1 MPa Mannitol)	-0.480	-0.588	-0.321	-0.544	-0.381	-0.840*	-0.667	-0.499	-0.932**	-0.410	-0.931	0.559	-0.954**	-0.525	-0.910**	0.916**	0.867**	0.827*	0.988**	0.058	1			
Na^+ flux of petiole (200mM NaCl)	-0.323	-0.796	-0.138	-0.322	-0.209	-0.656	-0.392	-0.315	-0.963**	-0.686	-0.919**	0.797	-0.921**	-0.546	-0.844*	0.747	0.878**	0.966**	0.906**	-0.136	0.944**	1		
Na^+ flux of petiole (0.75 MPa Mannitol)	0.073	-0.773	0.264	0.082	0.192	-0.310	-0.044	0.089	-0.790	-0.804*	-0.691	0.944**	-0.688	-0.259	-0.560	0.447	0.641	0.965**	0.750	-0.061	0.768	0.916**	1	
Na^+ flux of petiole (0.1 MPa Mannitol)	-0.387	-0.953**	-0.206	-0.293	-0.279	-0.551	-0.209	-0.344	-0.953**	-0.843*	-0.905**	0.838*	-0.879**	-0.724	-0.818*	0.600	0.867**	0.959**	0.719	-0.474	0.806*	0.936**	0.852**	1

"*" indicates significant correlation ($p < 0.05$); "**" indicates extremely significant correlation ($p < 0.01$).

International Journal of *Molecular Sciences*

MDPI

Supplementary Table S5. Principal component analysis of wild type and *PtEXPA6*-transgenic poplars of 717-1B4.

Index	PC1	PC2	PC3
Stem height growth	0.970	0.114	0.217
Stem diameter growth	0.930	0.342	0.136
Pn	0.975	-0.210	0.074
E	0.991	0.048	0.126
Cleaf	0.982	-0.017	-0.190
ETR	0.955	0.135	0.265
Y(Ⅱ)	0.979	0.049	0.199
Fv/Fm	0.811	0.483	0.330
POD activity	0.987	0.154	0.048
SOD activity	0.997	-0.064	-0.040
CAT activity	0.588	0.420	-0.691
Relative electrolyte leakage	-0.892	0.295	0.342
PODa2 relative expression level	0.964	0.263	-0.034
SOD [Cu-Zn] relative expression level	0.789	0.614	-0.029
CAT1 relative expression level	0.947	0.041	-0.319
Na^+ Content of Root	-0.980	0.187	-0.070
Na^+ Content of Stem	-0.846	0.461	-0.268
Na^+ Content of Leaf	-0.971	0.090	0.221
Na^+ flux of root (200mM NaCl)	-0.879	0.271	0.393
Na^+ flux of root (0.75 MPa Mannitol)	-0.912	0.409	0.034
Na^+ flux of root (0.1 MPa Mannitol)	-0.879	0.343	0.331
Na^+ flux of petiole (200mM NaCl)	-0.997	-0.069	-0.037
Na^+ flux of petiole (0.75 MPa Mannitol)	-0.777	0.379	-0.503
Na^+ flux of petiole (0.1 MPa Mannitol)	-0.978	-0.130	-0.160
Eigenvalues	20.337	1.949	1.714
Contribution rate (%)	84.737	8.120	7.144
Accumulative contribution rate (%)	84.737	92.856	100.000

Supplementary Table S6. Principal component analysis of measured traits of wild type and *PtEXPA6*-transgenic poplars of 84K.

Index	PC1	PC2	PC3
Stem height growth	0.725	0.638	0.259
Stem diameter growth	0.726	-0.557	0.402
Pn	0.580	0.764	0.282
E	0.702	0.713	0.006
Cleaf	0.638	0.716	0.282
ETR	0.882	0.411	-0.231
Y(Ⅱ)	0.642	0.632	-0.434
Fv/Fm	0.714	0.680	0.168
POD activity	0.973	-0.229	-0.026
SOD activity	0.486	-0.790	0.373
CAT activity	0.997	-0.075	-0.021
Relative electrolyte leakage	-0.500	0.865	-0.040
PODa2 relative expression level	0.994	-0.049	-0.096
SOD [Cu-Zn] relative expression level	0.823	0.179	0.540
CAT1 relative expression level	0.994	0.105	-0.037
Na^+ Content of Root	-0.891	-0.275	0.362
Na^+ Content of Stem	-0.990	0.062	-0.127
Na^+ Content of Leaf	-0.786	0.615	0.063
Na^+ flux of root (200mM NaCl)	-0.848	0.122	0.515
Na^+ flux of root (0.75 MPa Mannitol)	0.307	-0.126	0.943
Na^+ flux of root (0.1 MPa Mannitol)	-0.916	0.134	0.378
Na^+ flux of petiole (200 mM NaCl)	-0.886	0.418	0.200
Na^+ flux of petiole (0.75 MPa Mannitol)	-0.633	0.737	0.239
Na^+ flux of petiole (0.1 MPa Mannitol)	-0.877	0.454	-0.156
Eigenvalues	15.068	6.259	2.673
Contribution rate (%)	62.782	26.08	11.138
Accumulative contribution rate (%)	62.782	88.862	100
